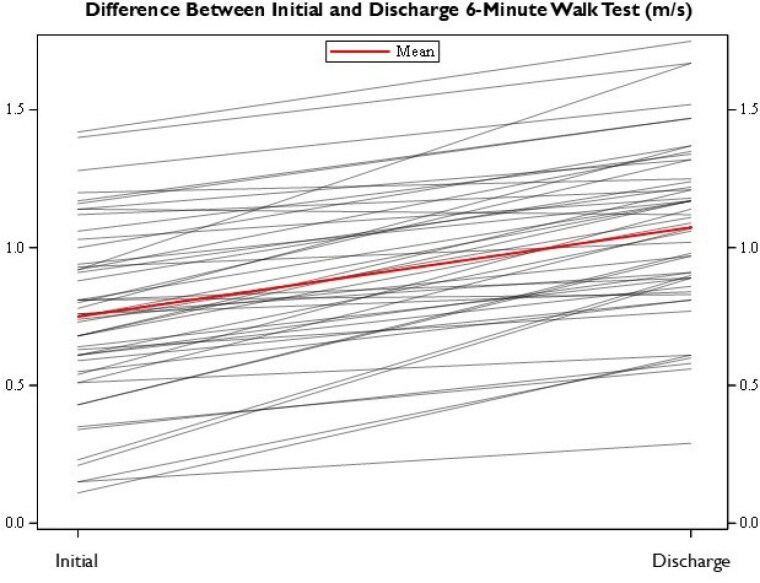# 693 Evaluating Post-Acute Burn Recovery via the 6-Minute Walk Test

**DOI:** 10.1093/jbcr/iraf019.322

**Published:** 2025-04-01

**Authors:** David Funk, Sarah Sabbatini, David Hill

**Affiliations:** Regional One Health Firefighter’s Burn Center; Regional One Health Firefighter’s Burn Center; Regional One Health Firefighter’s Burn Center

## Abstract

**Introduction:**

Analyzing gait speed via the 6-Minute Walk Test (6MWT) has been shown to be a reliable test for measuring overall functional capacity, strength, balance, and fall risk in patients recovering from various conditions including pulmonary disease, cardiovascular disease, stroke, and cancer. However, there has been limited research showing its effectiveness in measuring recovery over time in patients after burn injury. The objective of this study was to demonstrate the effectiveness of a burn-specific inpatient rehabilitation program. The primary hypothesis was that the 6MWT would be significantly improved between the post-acute initial and discharge measurement.

**Methods:**

Data was collected retrospectively from a single inpatient burn rehabilitation center. Patients performed two 6MWT at separate intervals during their inpatient rehab stay. The first measurement occurred as soon as deemed appropriate/safe by the therapist, and second just prior to discharge. Clinical history and demographic data were also recorded to account for other variables that may have affected the patient’s recovery and gait speed. SAS 9.4 was utilized for statistical analysis.

**Results:**

Fifty patients with a median age of 44 years (range 18 to 94) and total body surface area (TBSA) burned of 18% (range 2 to 64.5%) were included. All patients had either a lower extremity burn or donor site. Forty-seven patients received skin grafts during their acute inpatient admission. The average length of stay was 12.5 ± 5.4 days. There was a significant mean 118.44 meter (0.329 m/s) improvement in initial versus discharge 6MWT measurements (0.761 vs. 1.090 m/s). According to regression analysis, degree of improvement was negative correlated with the initial measurement, while controlling for TBSA injured and age of the patient.

**Conclusions:**

The 6MWT can be an effective outcome measure to evaluate overall burn recovery. Additionally, the significant improvement in functional outcome demonstrates the utility of an inpatient burn-specific rehabilitation program following acute inpatient treatment.

**Applicability of Research to Practice:**

Evaluating patients’ change in gait speed via the 6MWT, at the beginning and end of their inpatient burn rehab stay, is an effective way to demonstrate an increase in overall functional capacity and recovery post-burn.

**Funding for the Study:**

N/A